# Supplementary material for: De novo Design of All-atom Biomolecular Interactions with RFdiffusion3
Source: bioRxiv. 2025 Nov 19:2025.09.18.676967. Originally published 2025 Sep 18. Preprint. [Version 2] doi: 10.1101/2025.09.18.676967 (PMC12458353; doi:10.1101/2025.09.18.676967)
Supplement: 1 [file NIHPP2025.09.18.676967V2-supplement-1.pdf]

bioRxiv preprint doi: https://doi.org/10.1101/2025.09.18.676967; this version posted November 19, 2025. The copyright holder for this preprint (which was not certified by peer review) is the author/funder, who has granted bioRxiv a license to display the preprint in perpetuity. It is made available under aCC-BY 4.0 International license.

Supplementary Figures

bioRxiv preprint doi: https://doi.org/10.1101/2025.09.18.676967; this version posted November 19, 2025. The copyright holder for this preprint (which was not certified by peer review) is the author/funder, who has granted bioRxiv a license to display the preprint in perpetuity. It is made available under aCC-BY 4.0 International license.

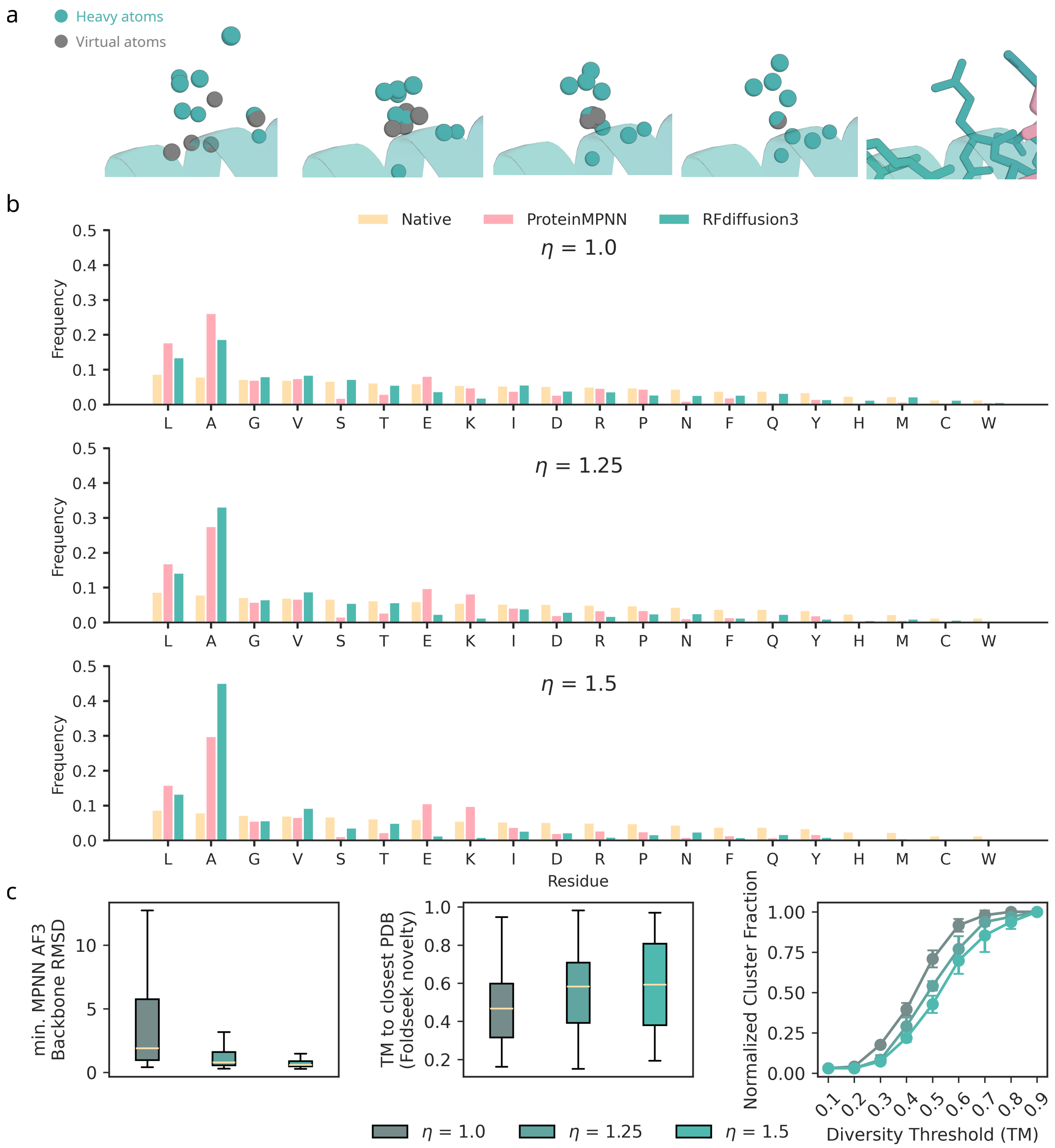

bioRxiv preprint doi: https://doi.org/10.1101/2025.09.18.676967; this version posted November 19, 2025. The copyright holder for this preprint (which was not certified by peer review) is the author/funder, who has granted bioRxiv a license to display the preprint in perpetuity. It is made available under aCC-BY 4.0 International license.

Fig. S1 ***(previous page)*****: Evaluation of sequence distribution of RFdiffusion3. a**, Diffusion of sequence via 14-atom representation. Additional virtual atoms are identified after diffusion as those placed on the C_β atom, and the residue identity can be decoded by the atom names of the heavy atoms. **b**, Sequence distributions of RFdiffusion3 for varying step scales (η), compared with the native (PDB) distribution, and the ProteinMPNN sequence fitted to the backbone. **c**, (left-to-right) Unconditional metrics of RFdiffusion3: designability of RFD3 with step scale (η); Novelty of RFD3 with step scale; Number of clusters under different TM thresholds as measure of diversity, normalized to the total number of clusters at TM$=1$. Overall, designability increases and diversity decreases with increasing step scale, forming a useful inference parameter for problems of interest. For this work, we opt to use $\eta = 1.5$ for benchmarks.

bioRxiv preprint doi: https://doi.org/10.1101/2025.09.18.676967; this version posted November 19, 2025. The copyright holder for this preprint (which was not certified by peer review) is the author/funder, who has granted bioRxiv a license to display the preprint in perpetuity. It is made available under aCC-BY 4.0 International license.

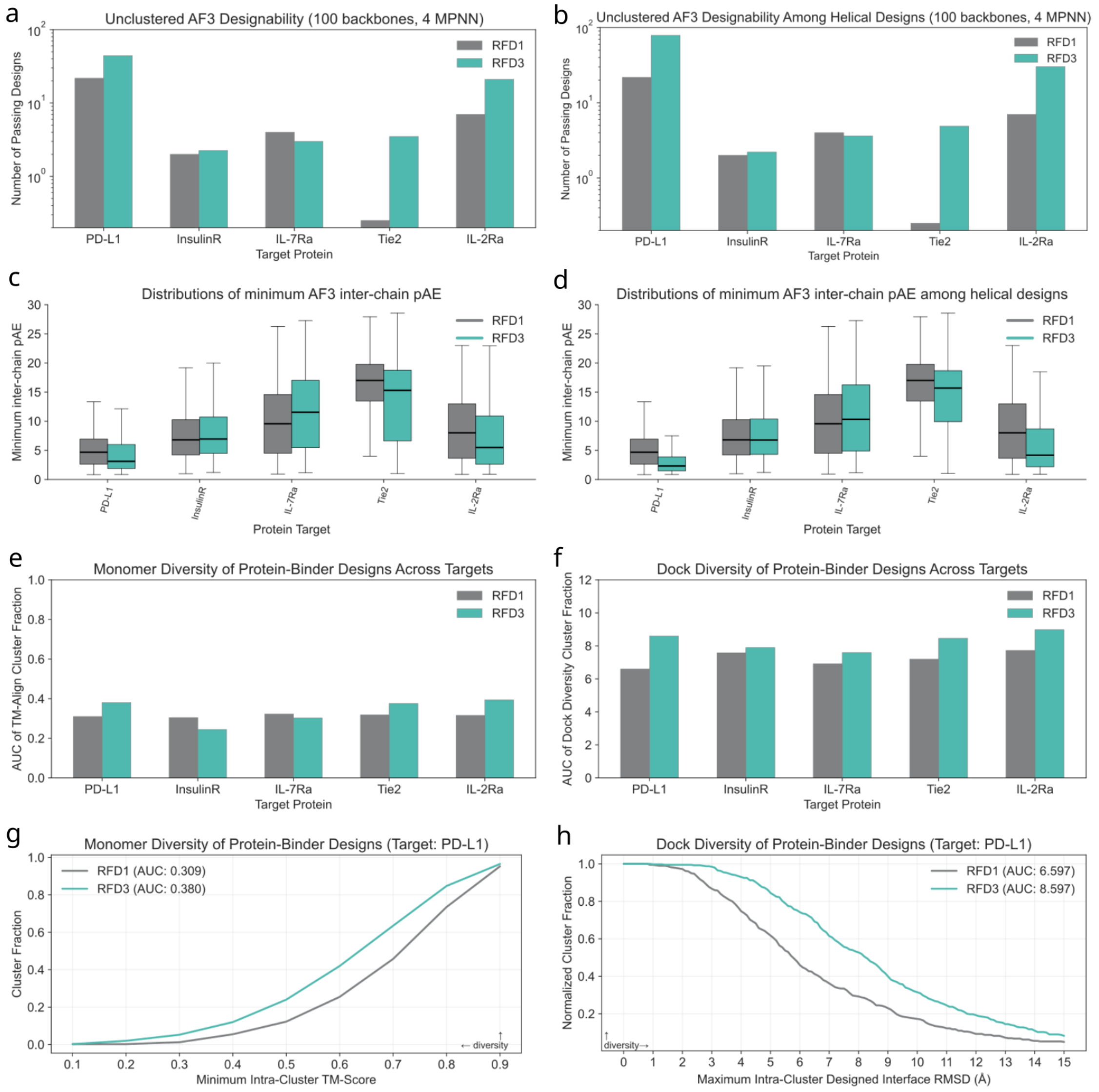

bioRxiv preprint doi: https://doi.org/10.1101/2025.09.18.676967; this version posted November 19, 2025. The copyright holder for this preprint (which was not certified by peer review) is the author/funder, who has granted bioRxiv a license to display the preprint in perpetuity. It is made available under aCC-BY 4.0 International license.

Fig. S2 ***(previous page)*****:** Designability and diversity of PPI Designs with RFD1 and RFD3. 400 backbones were designed for each of the five targets, then ProteinMPNN was used to design 4 sequences per backbone. **a,** Unclustered pass rate among all designs, normalized to 100 backbones (with 4 MPNNs) per target. Note that we use the stricter designability cutoffs introduced in [8] and detailed in the main text. **b,** Unclustered pass rate among primarily helical designs, defined as those with >75% helix content. This included all RFD1 designs and the following per-target counts with RFD3: PD-L1, 142 (36%); InsulinR, 359 (90%); IL-7Ra, 339, (85%); Tie2, 274 (69%); IL-2Ra, 143 (36%). As before, this plot is normalized to 100 backbones (with 4 MPNNs) per target. **c-d,** Distributions of the minimum AF3 inter-chain pAE among all designs and helical designs, respectively. Taken together, these results show that RFD3 significantly outperforms RFD1 when using the full set of designs, and that the advantage grows even further when subsetting to RFD1-style mostly-helical designs. Despite the higher pass rates among helical bundles, we believe it advantageous to have a model that is not limited to this fold type. **e,** AUC values for monomer diversity. For each target, 400 designed backbones were agglomeratively clustered at different TM-score thresholds using the "complete linkage" criterion, meaning that any given pair of elements in a cluster must have a TM-score above the threshold value. The metric used to compute the AUC is "cluster fraction", defined as the number of clusters divided by the number of examples, calculated at intervals of 0.1 between 0 and 1, exclusive. RFD3 shows higher monomer diversity than RFD1 across 3 of 5 targets. **f,** AUC values for the cluster fraction determined by a new metric, the "dock diversity". We define the dock diversity as the target-aligned backbone N, Cα, C RMSD between the 15 residues in the design that are closest to the target by Cα-Cα distance. The cluster thresholds are evenly spaced at intervals of 0.1 Å between 0 and 15, inclusive. As before, 400 designed backbones are clustered agglomeratively using the "complete linkage" criterion. RFD3 shows higher dock diversity than RFD1 on all targets. **g-h,** AUC curves for the 400 designs for PD-L1, displaying monomer diversity (**g**) and dock diversity (**h**).

bioRxiv preprint doi: https://doi.org/10.1101/2025.09.18.676967; this version posted November 19, 2025. The copyright holder for this preprint (which was not certified by peer review) is the author/funder, who has granted bioRxiv a license to display the preprint in perpetuity. It is made available under aCC-BY 4.0 International license.

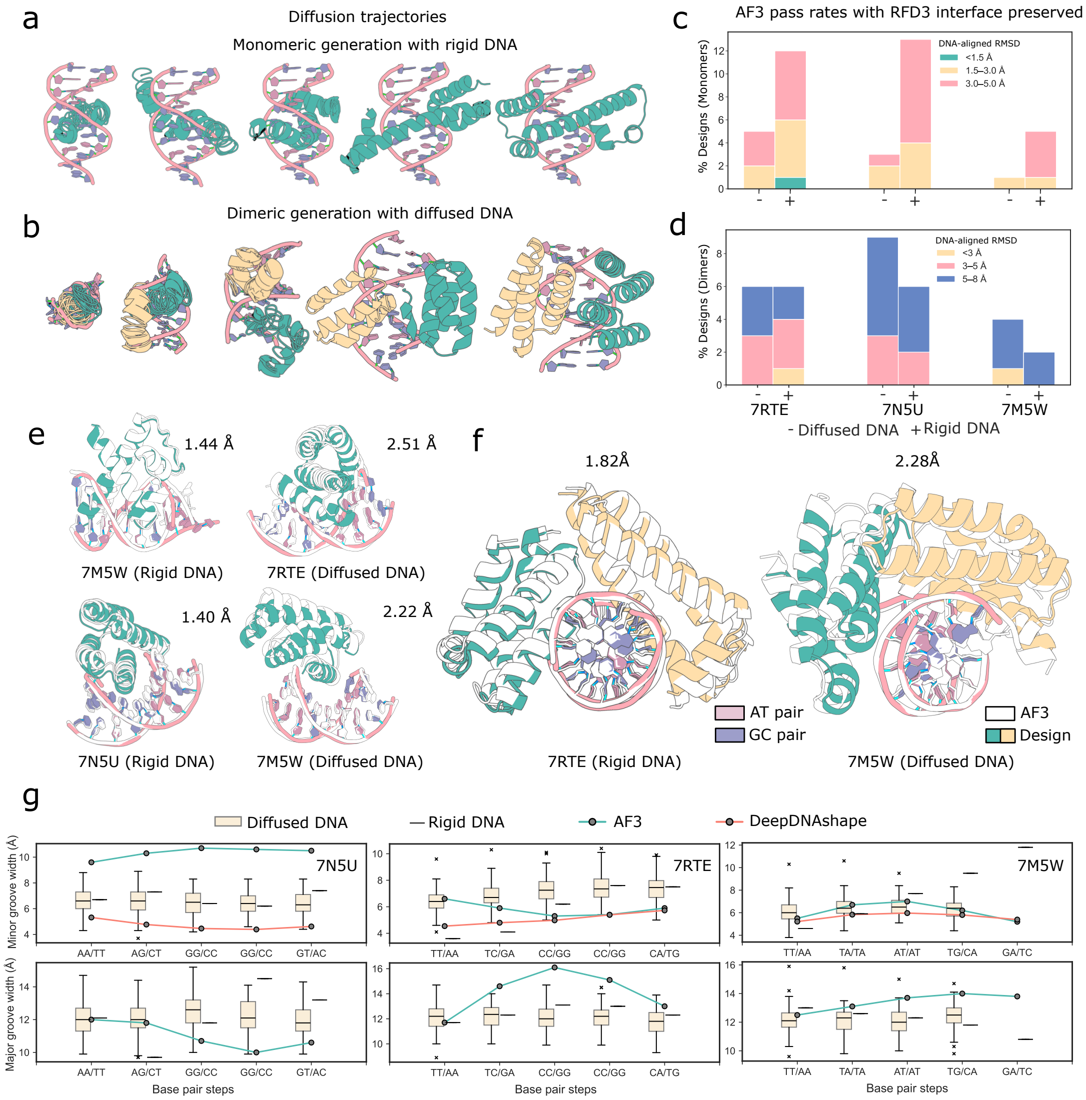

bioRxiv preprint doi: https://doi.org/10.1101/2025.09.18.676967; this version posted November 19, 2025. The copyright holder for this preprint (which was not certified by peer review) is the author/funder, who has granted bioRxiv a license to display the preprint in perpetuity. It is made available under aCC-BY 4.0 International license.

Fig. S3 ***(previous page)*****: Design of DNA interactions with RFdiffusion3. a,** Diffusion trajectory of monomeric de novo DNA binder generation using RFD3 against rigid DNA structure (from PDB ID: 7RTE). **b,** Diffusion trajectory of dimeric de novo DNA binder generation using RFD3 against diffused DNA (i.e. RFD3 generates DNA structure; DNA sequence from PDB ID: 7N5U). **c,** Percentage of agreement with AF3 prediction of RFD3 interface preserved (residues within 2-3.5 Å of DNA atoms)-LigandMPNN designs (monomeric) across three different targets, at different DNA-aligned RMSD ranges (<1.5 Å, 1.5-3 Å, 3-5 Å) and against both diffused and rigid DNA targets. **d,** Percentage of agreement with AF3 prediction of RFD3 interface preserved (residues within 2-3.5 Å of DNA atoms)-LigandMPNN sequences (dimeric) across three different targets, at different DNA-aligned RMSD ranges (<3 Å, 3-5 Å, 5-8 Å) and against both diffused and rigid DNA targets. **e,** Illustration of agreement with AF3 predictions for selected monomeric designs. **f,** Illustration of agreement with AF3 predictions for selected dimeric designs. **g,** RFD3 generated diverse protein-bound DNA conformations in the context of corresponding PDB (rigid) conformations and oracle predicted (AF3 and DeepDNAshape [58]) free DNA conformations. Major (bottom row) and Minor (top row) groove widths were computed using 3DNA-Analyze [59] for RFD3 generated (diffused and rigid target) and AF3 predicted structure of three different DNA target sequences. DeepDNAshape predicted minor groove width is also shown (averaged over consecutive bases to achieve base-pairstep values consistent with 3DNA-Analyze. Major groove width prediction was unavailable) Note: for 7M5W, output of 3DNA-analyze only contained values for 4 base-pair steps.

bioRxiv preprint doi: https://doi.org/10.1101/2025.09.18.676967; this version posted November 19, 2025. The copyright holder for this preprint (which was not certified by peer review) is the author/funder, who has granted bioRxiv a license to display the preprint in perpetuity. It is made available under aCC-BY 4.0 International license.

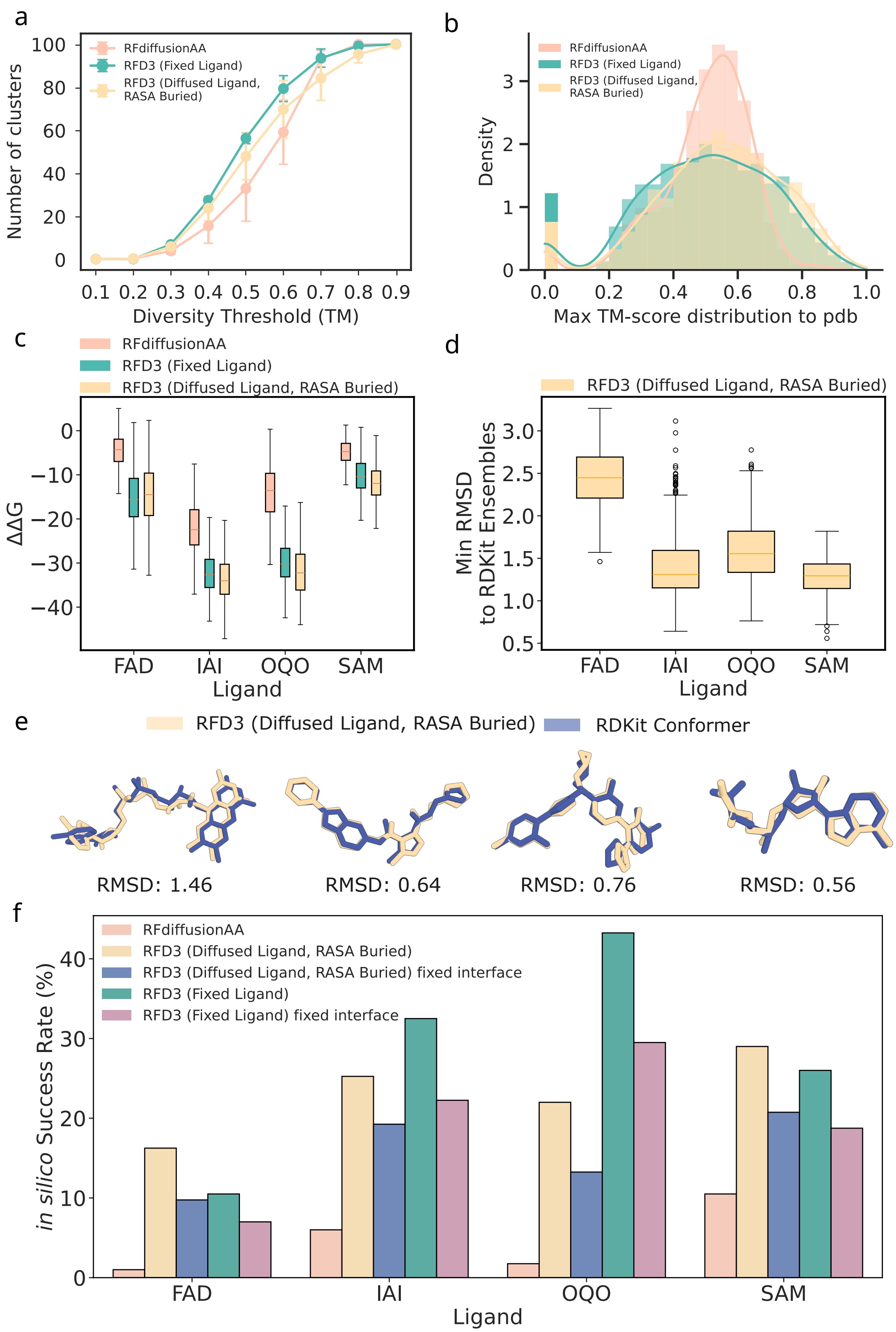

bioRxiv preprint doi: https://doi.org/10.1101/2025.09.18.676967; this version posted November 19, 2025. The copyright holder for this preprint (which was not certified by peer review) is the author/funder, who has granted bioRxiv a license to display the preprint in perpetuity. It is made available under aCC-BY 4.0 International license.

Fig. S4 ***(previous page)*****: Design of small-molecule binding proteins with RFdiffusion3**
a, Diversity of generated small molecule binders. Pairwise structural similarity among the designs is assessed using all-by-all TM-align. To quantify diversity, we performed agglomerative clustering with TM-score thresholds ranging from 0 to 1 and reported the number of clusters at each threshold. For comparison, the same analysis was applied to 400 designs generated by RFdiffusion All-Atom for each target. Results across four ligands were averaged, with error bars representing variability across targets. Overall, RFdiffusion3 generated more diverse structures than RFdiffusion All-Atom. **b,** For the same set of 400 RFdiffusion3 designs described in *a*, we used FoldSeek to identify the closest structural neighbor in the PDB for each binder and recorded its TM-score. We then evaluated novelty by counting the number of designs whose maximum TM-score (i.e., similarity to the closest PDB structure) falls below thresholds ranging from 0 to 1. This quantifies how distinct the generated binders are relative to known protein structures. The analysis was performed for each method, aggregated across all four ligands. **c,** We evaluated the binding energies of the designs described in *a* using Rosetta, applying the $\text{DDG}_{\text{norepack}}$ metric to assess binding quality. This metric estimates the change in binding free energy ($\Delta\Delta G$) between the bound and unbound states of a complex, without side-chain repacking during the calculation. Lower values indicate stronger binding. Outliers have been excluded to improve the clarity of the visualization. **d,** RMSD distribution between RFdiffusion3-generated diffused ligands and RDKit conformers. For each ligand target, 50 conformers were generated using RDKit. For each design under the diffused-ligand setting, we computed the RMSD between the generated ligand and all conformers and reported the minimum RMSD. **e,** Representative structures of RFdiffusion-generated diffused ligands showing the smallest RMSD to RDKit-generated conformers. **f,** Comparison of *in silico* designability across different settings. In addition to the standard benchmark, where LigandMPNN is used to redesign the entire binder sequence, we also assessed designability under a fixed-interface setting, in which interface residue identities were preserved and only the remaining regions were redesigned with LigandMPNN. The ligand–protein interface is defined as protein residues within 2–3.5 Å of any ligand atom. All evaluations use 400 backbones and 8 sequences per design, and the shared inference parameters of step scale $\eta = 1.5$, noise level $\gamma_0 = 0.6$, and 200 denoising steps (used throughout this work unless otherwise specified.) Finally, diffused-ligand binders were generated with the RASA condition set to buried and a CFG scale of 2 to guide the creation of ligand binding pockets.

bioRxiv preprint doi: https://doi.org/10.1101/2025.09.18.676967; this version posted November 19, 2025. The copyright holder for this preprint (which was not certified by peer review) is the author/funder, who has granted bioRxiv a license to display the preprint in perpetuity. It is made available under aCC-BY 4.0 International license.

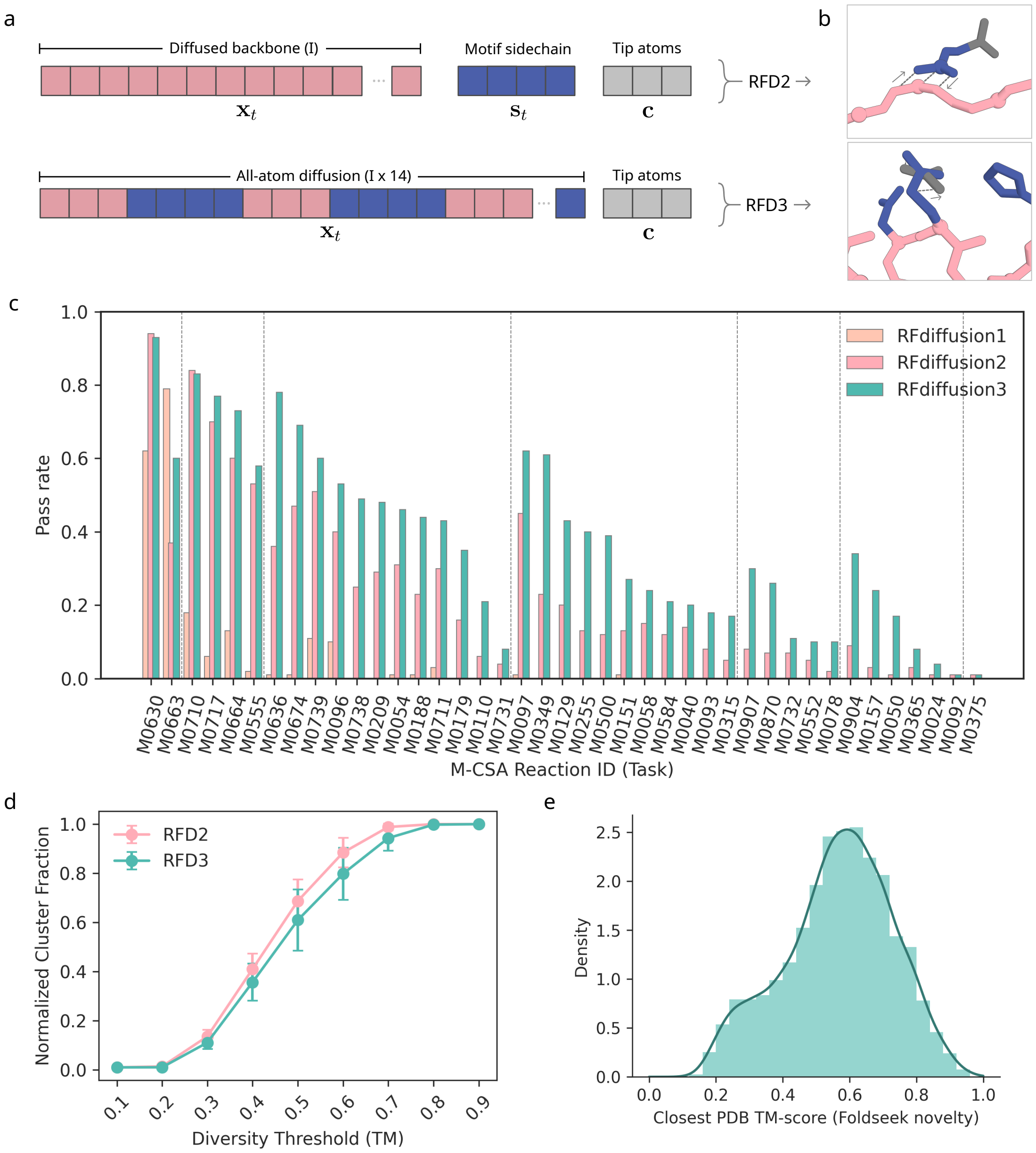

bioRxiv preprint doi: https://doi.org/10.1101/2025.09.18.676967; this version posted November 19, 2025. The copyright holder for this preprint (which was not certified by peer review) is the author/funder, who has granted bioRxiv a license to display the preprint in perpetuity. It is made available under aCC-BY 4.0 International license.

Fig. S5 ***(previous page)*****: Design of enzymes with RFdiffusion3.** **a,** Atomic Motif Enzyme Benchmark of RFdiffusion variants. For benchmark details see section Section 3.5. **b,** Tokenization of unindexed atoms with RFdiffusion2 involves explicit tokenization of the motif sidechains belonging to the (fixed condition) tip atoms. In contrast, RFdiffusion3 natively diffuses sidechains, making explicit tokenization of motif sidechains unnecessary. **c,** Diversity analysis of RFD3 against RFD2. Diversity analysis includes full generated ensemble. Error bars indicate the variance across the 41 different AME tasks at a particular diversity threshold. **d,** Novelty analysis of outputs on the AME benchmark. RFD3 produces a significant number of novel folds (signified by high density between TM 0.2 and 0.4.)

bioRxiv preprint doi: https://doi.org/10.1101/2025.09.18.676967; this version posted November 19, 2025. The copyright holder for this preprint (which was not certified by peer review) is the author/funder, who has granted bioRxiv a license to display the preprint in perpetuity. It is made available under aCC-BY 4.0 International license.

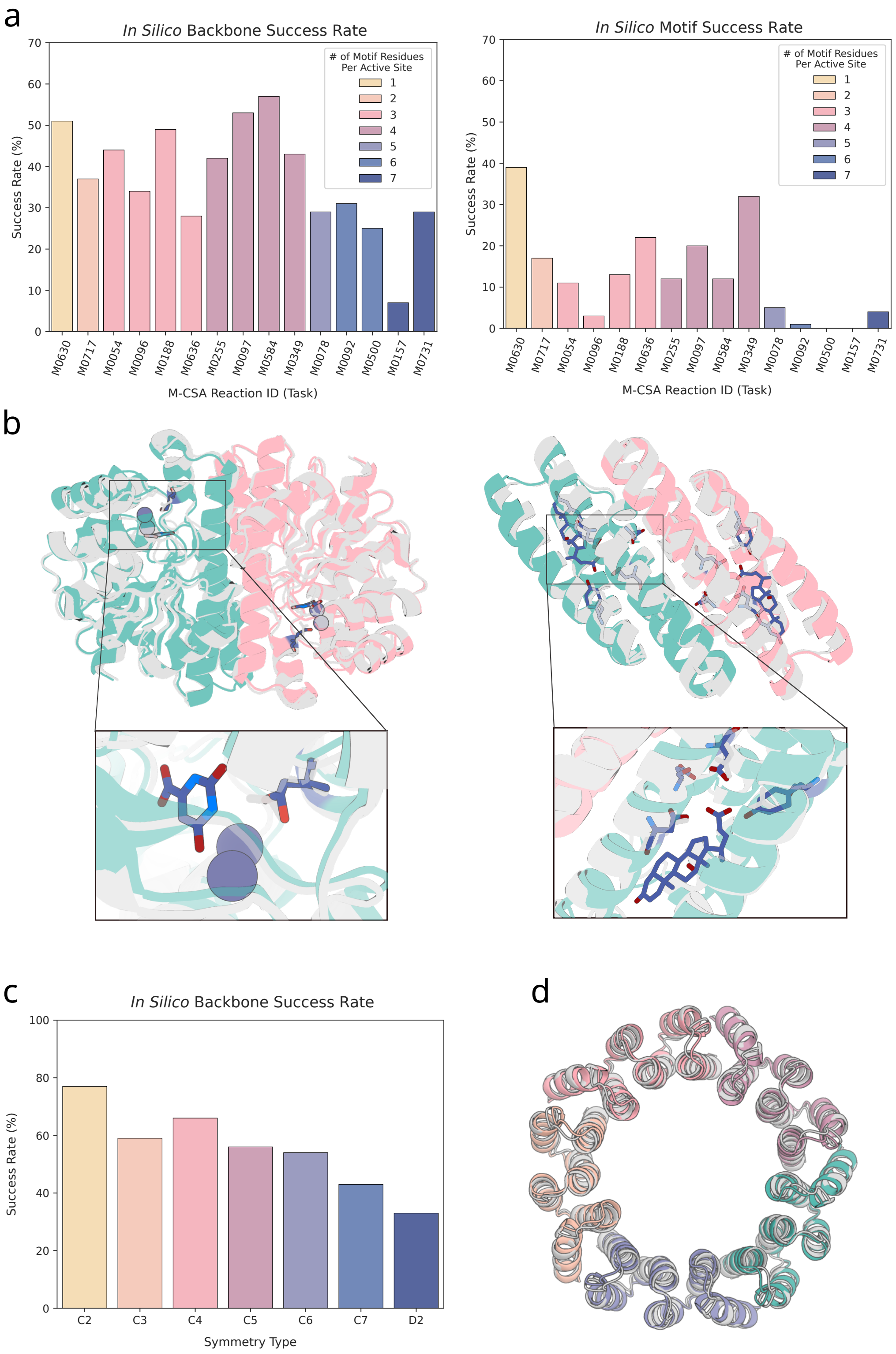

bioRxiv preprint doi: https://doi.org/10.1101/2025.09.18.676967; this version posted November 19, 2025. The copyright holder for this preprint (which was not certified by peer review) is the author/funder, who has granted bioRxiv a license to display the preprint in perpetuity. It is made available under aCC-BY 4.0 International license.

Fig. S6 ***(previous page)*****: Design of symmetric enzymes with RFdiffusion3. a,** *In silico* success rates for backbones on a subset of the AME benchmark. (left) Success rate for backbones of complexes. (right) Success rate of active site residues in an asymmetric unit (ASU) on top of the complex backbone success. **b,** Example outputs from **a**. (left) M0630 C2 enzyme with complex backbone Cα RMSD of 0.84 Åand motif all-atom RMSD of 0.46 Å(AF3). (right) M0349 C2 enzyme with complex backbone Cα RMSD of 1.08 Å and ASU motif all-atom RMSD of 0.66 Å. **c,** *In silico* success rate for backbones on symmetry-constrained diffusion without motifs. **d,** Example output for C5 symmetry with Cα RMSD of 0.91 Å.

bioRxiv preprint doi: https://doi.org/10.1101/2025.09.18.676967; this version posted November 19, 2025. The copyright holder for this preprint (which was not certified by peer review) is the author/funder, who has granted bioRxiv a license to display the preprint in perpetuity. It is made available under aCC-BY 4.0 International license.

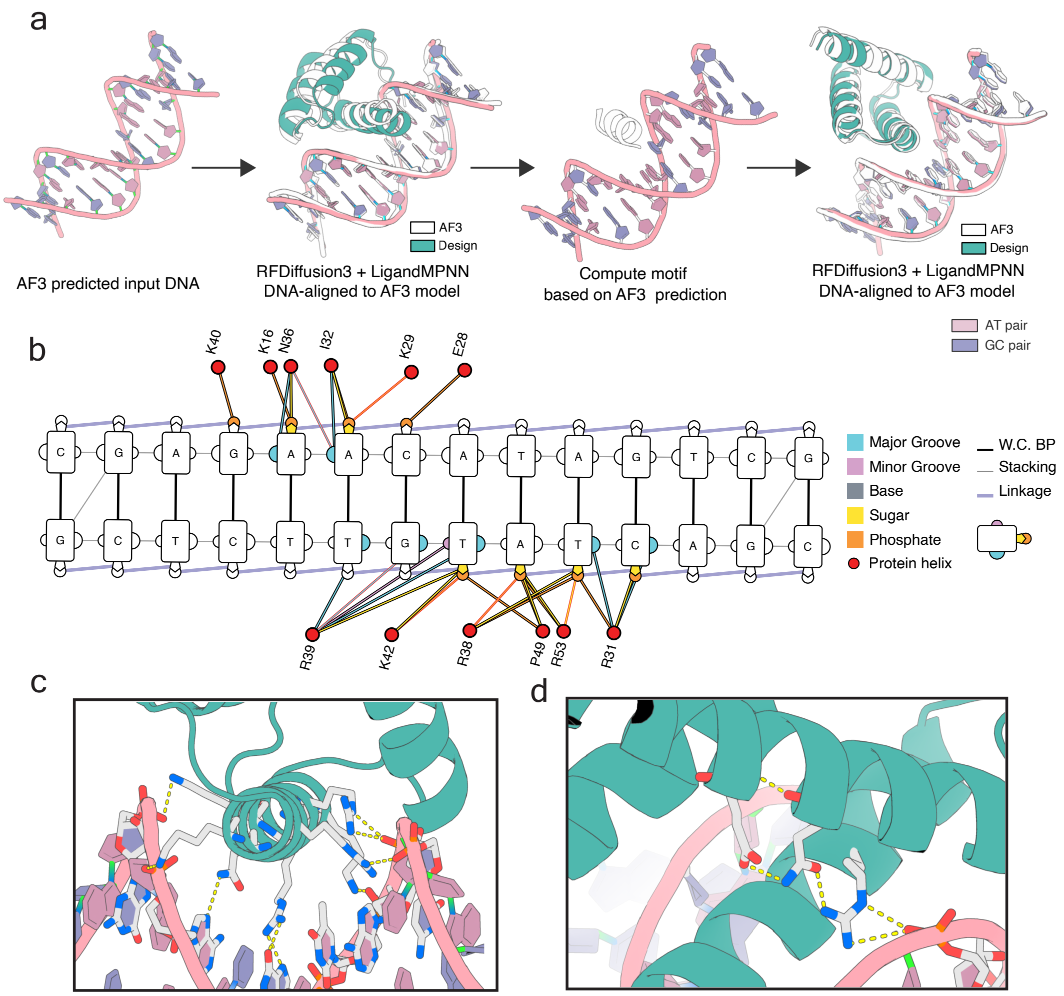

bioRxiv preprint doi: https://doi.org/10.1101/2025.09.18.676967; this version posted November 19, 2025. The copyright holder for this preprint (which was not certified by peer review) is the author/funder, who has granted bioRxiv a license to display the preprint in perpetuity. It is made available under aCC-BY 4.0 International license.

Fig. S7 ***(previous page)*****: Design of DNA binders with RFdiffusion3. a,** Schematic representation of the DNA-binder design pipeline used to design DBRFD3. Full details in Section 4.2. **b,** Protein-DNA contact map of DBRFD3 generated using DNAproDB [60]. Interaction lines indicated with bold red borders denote hydrogen bonding. **c,** DBRFD3 recognizes DNA through multiple major groove and backbone interactions with ASN36 (N36) and ARG39 (R39) performing major groove hydrogen bond interactions, to recognize adenine and guanine bases, respectively. **d,** Hierarchical supportive hydrogen bond interactions observed in DBRFD3 (usually uncommon in *de novo* designs). The positively charged residue ARG38 interacts with the negatively charged phosphate moiety. The neutral residue GLN56 supports ARG38. GLN56 is, in turn, supported by negatively charged GLU60.

bioRxiv preprint doi: https://doi.org/10.1101/2025.09.18.676967; this version posted November 19, 2025. The copyright holder for this preprint (which was not certified by peer review) is the author/funder, who has granted bioRxiv a license to display the preprint in perpetuity. It is made available under aCC-BY 4.0 International license.

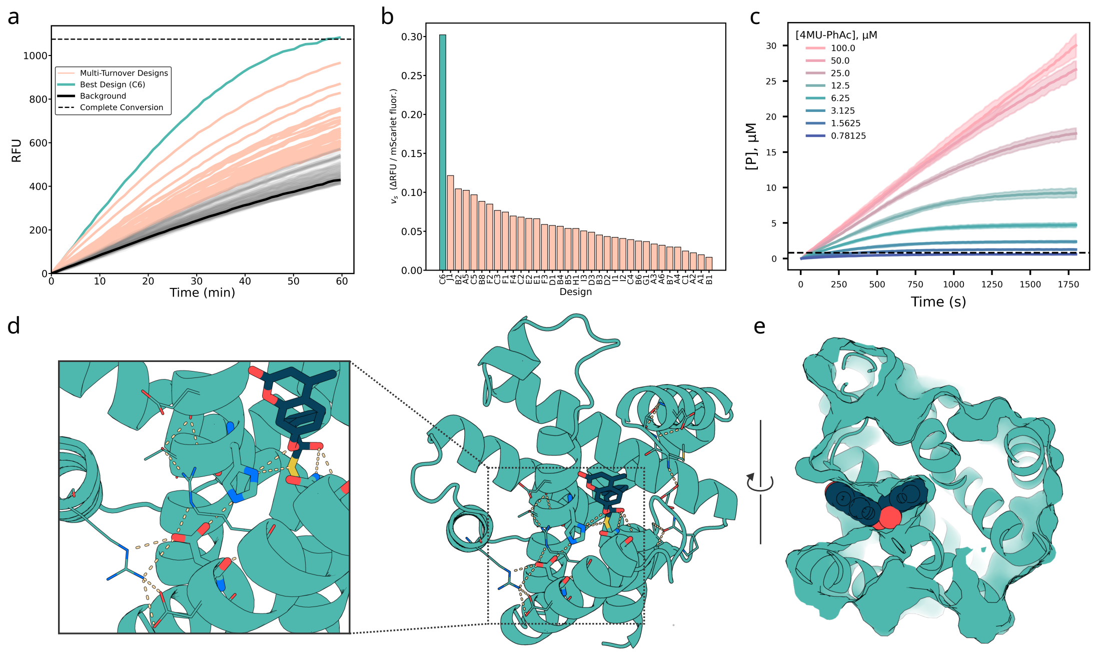

bioRxiv preprint doi: https://doi.org/10.1101/2025.09.18.676967; this version posted November 19, 2025. The copyright holder for this preprint (which was not certified by peer review) is the author/funder, who has granted bioRxiv a license to display the preprint in perpetuity. It is made available under aCC-BY 4.0 International license.

Fig. S8 ***(previous page)*****: Experimental characterization and structural analysis of** ***de novo*** **cysteine hydrolase designs.** **a,** IVTT screen of 190 designs using 4MU–PhAc substrate. Representative fluorescence traces (RFU) are shown for background controls (black), multi-turnover designs exceeding the 10% conversion threshold (cyan), and the best design, C6 (orange). The dashed line indicates full conversion. **b,** Normalized steady-state rates (background-subtracted and scaled by mScarlet fluorescence) for the 35 multi-turnover designs, grouped by scaffold family and indexed numerically. C6 is highlighted in orange. **c,** Michaelis–Menten kinetics of purified C6 measured in E. coli expression. Product formation is shown across eight substrate concentrations (mean ± s.d., n = 3). Dashed line shows enzyme concentration in the reaction well (0.8 μM) **d,** Active site of C6 (AF3 prediction). (left) zoomed-in view showing pre-organized hydrogen-bonding network at the active site: the Asp general base locked in place by dual hydrogen bonds from two arginines, themselves supported by Asp and Thr a nearby helices. (right) Global view of scaffold with catalytic residues and substrate in blue, and the hydrogen-bonding networks highlighted. **e,** AF3 prediction of C6 in complex with the modeled tetrahedral intermediate (TI1), showing shape-complementary pocket geometry and correct orientation of the 4MU leaving group toward solvent.

bioRxiv preprint doi: https://doi.org/10.1101/2025.09.18.676967; this version posted November 19, 2025. The copyright holder for this preprint (which was not certified by peer review) is the author/funder, who has granted bioRxiv a license to display the preprint in perpetuity. It is made available under aCC-BY 4.0 International license.

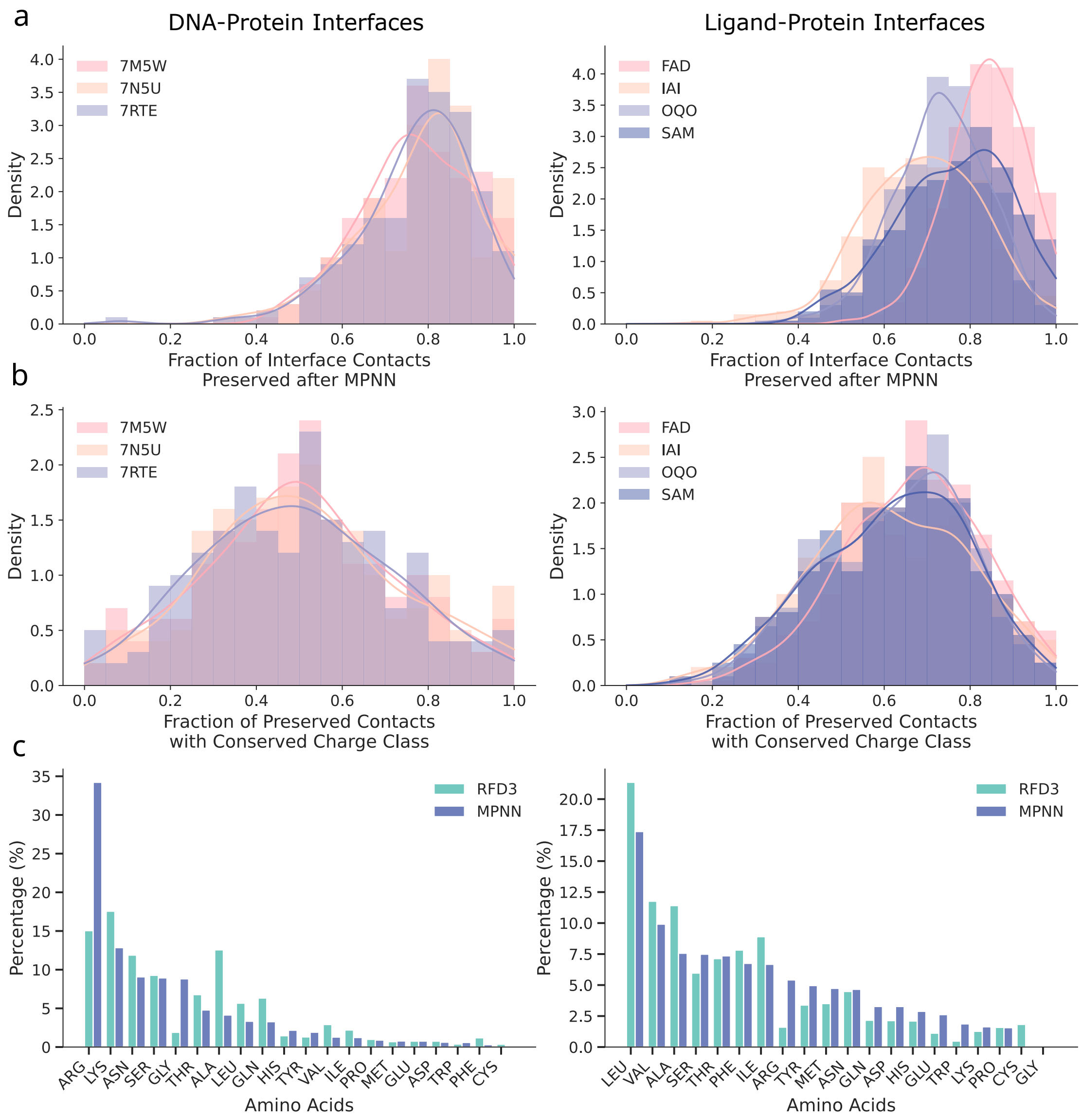

bioRxiv preprint doi: https://doi.org/10.1101/2025.09.18.676967; this version posted November 19, 2025. The copyright holder for this preprint (which was not certified by peer review) is the author/funder, who has granted bioRxiv a license to display the preprint in perpetuity. It is made available under aCC-BY 4.0 International license.

Fig. S9 ***(previous page)*****: Analysis of interface residue composition between RFdiffusion3 generated sequences, LigandMPNN sequences and native sequences. a**, Fraction of interface residue contacts preserved after MPNN. For each design case, we generated 400 structures using RFdiffusion3. For DNA targets, ProteinMPNN was used to design 4 sequences per structure, and for ligand targets, LigandMPNN was used to design 8 sequences per structure. Interface residues were identified in both the RFdiffusion3 designs and the corresponding MPNN-designed structures using a 2–3.5 Ådistance threshold. We then computed, for each design, the fraction of interface contacts that were preserved before and after MPNN sequence design, and reported the average value across all the MPNN-packed structures for each design. **b**, Fraction of preserved interface-contact residues that also retain their charge class (positive, negative, or neutral). Using the same setting as in *a* we measured, among all preserved interface contacts, the fraction where the contacting residues share the same charge class before and after MPNN. **c**, Distribution of amino acids at the interface. Interface residues were defined as protein residues within 2–3.5 Åof any DNA or ligand atom.
